# A Hydrazine–Hydrazone Adamantine Compound Shows Antimycobacterial Activity and Is a Probable Inhibitor of MmpL3

**DOI:** 10.3390/molecules27207130

**Published:** 2022-10-21

**Authors:** Julien Briffotaux, Yanji Xu, Wei Huang, Zhen Hui, Xiao Wang, Brigitte Gicquel, Shengyuan Liu

**Affiliations:** 1 Department of Tuberculosis Control and Prevention, Shenzhen Nanshan Center for Chronic Disease Control, 7 Huaming Road, Shenzhen 518054, China; 2 Bacteriology & Antibacterial Resistance Surveillance Laboratory, Shenzhen Institute of Respiratory Diseases, Shenzhen People’s Hospital, The Second Clinical Medical College, Jinan University, The First Affiliated Hospital, Southern University of Science and Technology, No 1017 Dongmen North Road, Shenzhen 518020, China; 3 Mycobacterial Genetics Unit, Institut Pasteur, 25 Rue du Docteur Roux, 75724 Paris, France

**Keywords:** tuberculosis, MmpL3, drug screening, small molecules, drug resistance

## Abstract

Tuberculosis remains an important cause of morbidity and mortality throughout the world. Notably, an important number of multi drug resistant cases is an increasing concern. This problem points to an urgent need for novel compounds with antimycobacterial properties and to improve existing therapies. Whole-cell-based screening for compounds with activity against *Mycobacterium tuberculosis* complex strains in the presence of linezolid was performed in this study. A set of 15 bioactive compounds with antimycobacterial activity in vitro were identified with a minimal inhibitory concentration of less than 2 µg/mL. Among them, compound **1** is a small molecule with a chemical structure consisting of an adamantane moiety and a hydrazide–hydrazone moiety. Whole genome sequencing of spontaneous mutants resistant to the compounds suggested compound **1** to be a new inhibitor of MmpL3. This compound binds to the same pocket as other already published MmpL3 inhibitors, without disturbing the proton motive force of *M. bovis* BCG and *M. smegmatis*. Compound **1** showed a strong activity against a panel ofclinical strains of *M. tuberculosis* in vitro. This compound showed no toxicity against mammalian cells and protected *Galleria mellonella* larvae against *M. bovis* BCG infection. These results suggest that compound **1** is a promising anti-TB agent with the potential to improve TB treatment in combination with standard TB therapies.

## 1. Introduction

Tuberculosis (TB), caused by *Mycobacterium tuberculosis*, is among the major infectious diseases in humans, leading to 1.5 million deaths in 2020 [1]. The current treatment for drug-sensitive TB consists of a combination of isoniazid, rifampicin, ethambuthol, and pyrazinamide, but requires several months under strict observation. Moreover, despite efforts to end the epidemic, TB persists and antibiotic-resistant strains of *M. tuberculosis* continue to emerge. In this context, it is necessary to continue efforts to develop new molecules with antimycobacterial properties and facilitators or enhancers of approved antibiotics [2,3].

The World Health Organization recommendations for the treatment of drug-resistant and multi-drug resistant (MDR) TB include fluoroquinolones, bedaquiline, and linezolid [4,5]. Bedaquiline is a new drug that was approved in 2012 for the treatment of multidrug-resistant tuberculosis with the ability to inhibit mycobacterial F1F0 ATP synthase [6]. Linezolid is a protein synthesis inhibitor that binds to the peptidyl transferase center in the active site of 50S ribosomal subunits [7]. First used for the treatment of infections by resistant Gram-positive bacteria, linezolid is also effective in curing patients with multiple-drug resistant (MDR) and extensive drug-resistant (XDR) TB [8]. There are other anti-TB drugs that can be used for second-line regimens, such as fluoroquinolones, betalactams, para-aminosalicylic acid, Clofazimine, and Cycloserine. These drugs can be used in varying combinations depending on the circumstances. However, most drugs that are used for MDR TB treatments show side effects making their long-term utilization difficult. In addition, *M. tuberculosis* clinical isolates resistant to bedaquiline were found after its approval, thus showing the need for other drugs in certain cases. The resurgence of MDR TB due to the COVID-19 pandemic makes it even more necessary to identify new molecules and shorten the duration of treatment [9].

The use of drug combinations with a compound that would increase the activity of linezolid and thus reduce the amount required is among possible strategies to reduce the toxicity of linezolid. Combination therapeutics are also excellent strategies to limit the emergence of antibiotic resistance and thus extend the life of antibiotics [10]. We have previously developed a whole-cell screening method coupled with a resazurin reduction as a marker of cellular viability to identify molecules with anti-mycobacterial activity [11]. In order to identify the potential target or the mode of action of the hit compounds, it is possible to use the genome-wide transcriptional response of *Mycobacterium* exposed to the compound [12] or the whole-genome sequencing of resistant mutants [13].

In this study, we undertook whole-cell screening in the presence of subinhibitory concentrations of linezolid coupled with whole-genome sequencing of spontaneous resistant mutants to identify synergistic or compatible combination treatments and their target. Among 52,000 chemical compounds of various families, 15 compounds similar to or different from known antibiotics were found with a minimal inhibitory concentration <2 µg/mL. Here, we describe one small molecule with a novel structure that targets the essential lipid transmembrane transporter MmpL3, required for the export of the lipid trehalose monomycolate (TMM) [14,15]. Then, the toxicity of this compound to mammalian cells was determined, as well as its efficacy in the therapy of experimentally *M. bovis* BCG infected *Galleria* larvae.

## 2. Results and Discussion

### 2.1. Screening of the Chemical Library and Identification of Compound **1**

We performed a high-throughput screen for compounds against the growth of *M.* bovis BCG, as a representative of *M. tuberculosis* complex strains, in the presence of 0.15 µg/mL linezolid (a non-inhibitory concentration that represents a quarter of the minimal inhibitory concentration [MIC]) in 7H9 medium to identify compounds that may enhance the antimycobacterial activity of linezolid. A chemical library of 52,000 compounds was screened against the growth of *M. bovis* BCG assessed using the resazurin colorimetric assay. This library was screened at 2 μg/mL (average concentration of 5 to 6 μM). Compounds identified as active were then tested for MIC. We identified 224 active compounds during the first screening, 15 of them with a MIC < 2 µg/mL. After eliminating certain compounds already published by others, we selected active compounds for further investigation. Here, we focus on the results obtained with compound **1**.

**Figure 1:**
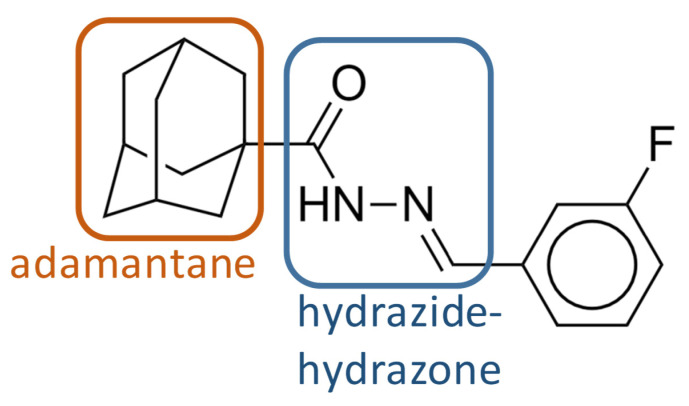
Compound 1 identified during the screening. The adamantane and hydrazide–hydrazone moieties are indicated.

Compound **1** has an adamantane moiety and a hydrazide–hydrazone moiety (Figure 1). The adamantane molecule consists of three connected cyclohexane rings. Adamantane derivatives, such as amantadine, have been found to have antiviral activity [16]. Adamantylureas, such as AU1235 [17] and SQ109 [18], were previously identified as a group of compounds that are active against *M. tuberculosisi* as potential inhibitors of MmpL3. SQ109 has completed phase 2 clinical trials [19,20]. Hydrazide–hydrazone derivatives have been found to have biological activity, such as antimicrobial [21,22,23], antituberculosis [24,25,26], and anticancer [27] drugs.

We examined the antimicrobial activities of compound **1** by determining the MIC and minimal bactericidal concentration (MBC) for various mycobacterial species (Table 1). The MBC was defined as the lowest concentration required to kill 99.9% of bacteria. Compound **1** showed an MIC of 0.2 µg/mL for *M. tuberculosis*, 0.3 µg/mL for *M. bovis* BCG, 1.5 µg/mL for *M. smegmatis*, and 12.5 µg/mL for *M. abscessus* and *M. marinum*. It showed an MBC of 0.6 µg/mL for *M. bovis* BCG and 3 µg/mL for *M. smegmatis*. We also carried out MIC testing of compound **1** on *S. aureus* and *E. coli* but there was no inhibition at concentrations up to 25 µg/mL, showing that the activity of compound **1** is likely specific to mycobacteria.

**Figure 2:**
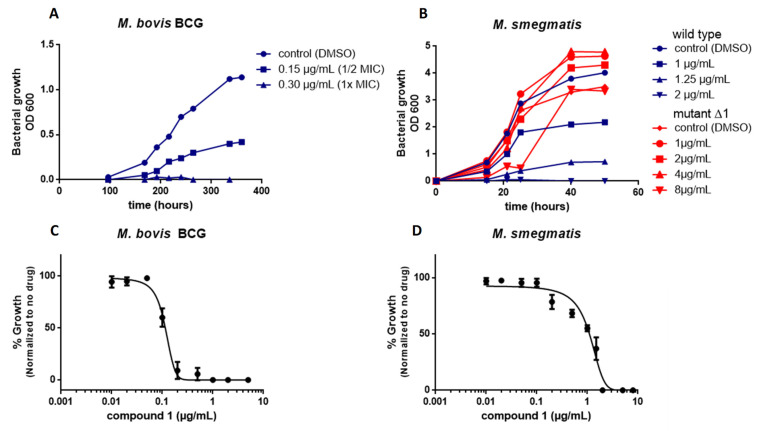
Compound 1 inhibits the growth of *M. bovis* BCG and *M. smegmatis* in vitro. All cultures were performed in 7H9 medium. (**A**) Growth curve of *M. bovis* BCG in the presence of various concentrations of compound **1**. (**B**) Growth curve of wild type *M. smegmatis* (blue) and the *M. smegmatis* resistant mutant Δ1 (red) in the presence of various concentrations of compound **1**. (**C**) Dose–response curves for the inhibition of *M. bovis* BCG by compound **1**. (**D**) Dose–response curves for the inhibition of *M. smegmatis* by compound **1**.

**Figure 3:**
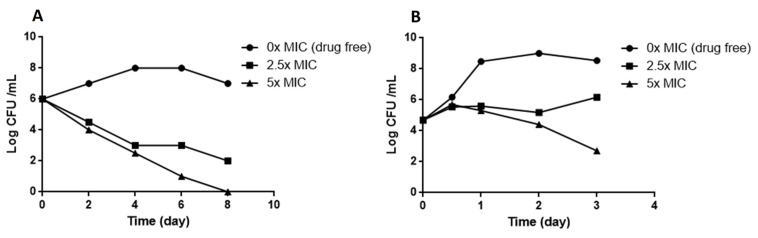
Kill kinetics for compound **1**. Exponential cultures of *M. bovis* BCG (**A**) and *M. smegmatis* (**B**) were exposed to various concentrations of compound **1** in 7H9 medium and the number of viable bacteria were determined by serial dilution and plating onto agar 7H11 plates at the indicated times. The control (drug free) was 0.5% DMSO.

We confirmed the antimycobacterial activity of compound **1** by kinetic growth assays. Up to 1× the MIC inhibited the growth of *M. bovis* BCG and *M. smegmatis* (Figure 2A,B). Compound **1** inhibited the growth of *M. smegmatis* and *M. bovis* BCG in a dose-dependent manner (Figure 2C,D). We tested whether compound **1** has a bacteriostatic or a bactericidal effect by culturing exponential phase *M. smegmatis* and *M. bovis* BCG in liquid medium in the presence of various concentrations of compound **1** as a time-kill assay (Figure 3). Compound **1** was rapidly bactericidal at 2.5× MIC (0.75 µg/mL) against *M. bovis* BCG and the effect was greater at a higher concentration (5× MIC or 1.5 µg/mL) (Figure 3A). This result is consistent with the observed MBC (0.6 µg/ml). Compound **1** showed only moderate bactericidal activity against *M. smegmatis* at 5× MIC (7.5 µg/mL) (Figure 3B).

### 2.2. Compound **1** Shows Strong Activity against Clinical Isolates of M. tuberculosis

We further evaluated the activity of compound **1** against *M. tuberculosis* H37Rv and a panel of clinical isolates of *M. tuberculosis* including sensitive (S17, S23, S31), rifampicin-resistant (M9, M20, M34), and MDR strains (M7). Information on the genes mutated in the drug-resistant strains is provided in Supplementary Information (Table S1). The MIC of compound **1** was low against both sensitive and resistant clinical strains (Table 2). The susceptibility to the drug of six of the clinical strains fell between 0.2 and 0.78 µg/mL. Only strain M20 showed a slightly higher MIC of 1.56 µg/mL for an unknown reason. These results suggest that compound **1** is a good candidate for the treatment of sensitive and drug-resistant *M. tuberculosis* strains.

### 2.3. Combination of Compound **1** with Linezolid In Vitro

A checkerboard method assay was used to identify the drug interaction of compound **1** with linezolid. Combinations that result in a fractional inhibitory concentration index (FICI) ≤ 0.5 are considered to be synergistic and those that result in a 0.5 < FICI ≤ 1 are considered to be additive. Compound **1** showed a synergistic effect in combination with linezolid against *M. bovis* BCG and *M. smegmatis*, with FICIs of 0.50 and 0.28, respectively, whereas there was an additive effect against *M. tuberculosis* H37Rv, with a FICI of 0.68 (Table 3).

### 2.4. Mutations in MmpL3 Confer Resistance to Compound **1**

We isolated spontaneous *M. smegmatis* mutants resistant to compound **1** on 7H11 solid medium to identify its target. The observed rate of spontaneous mutations was approximately 10^8^ per bacterium. Mutants were then tested for resistance in liquid and solid media. The MIC of *M. smegmatis* mutants resistant to compound **1** was 16-times higher (25µg/mL) than that of the wild type strain. However, the isolation of resistant *M. bovis* BCG mutants was unsuccessful.

DNA isolated from three independent *M. smegmatis* mutants was subjected to whole-genome sequencing. Single nucleotide polymorphisms (SNPs) were identified (Table 4) and confirmed by PCR amplification and sequencing.

Three independent mutants were found to have the same missense mutations in *mmpL3* (*MSMEG_0250*), resulting in the conversion of phenylalanine to leucine at position 649 (F649L). This mutation corresponds with mutation F644L, previously described in *M. tuberculosis* and isolated for other MmpL3 inhibitors with different chemical structures [13,28,29,30,31] and is located in the center of the transmembrane domain of MmpL3. The presence of various polymorphisms at other loci strongly support that the isolated mutants are independent.

We next determined whether the point mutation in MmpL3 affects the growth of *M. smegmatis* in the absence or presence of compound **1** by comparing the growth curves of mutant Δ1 to that of the wild type parental strain. The mutation did not affect growth in 7H9 medium and the Δ1 mutant tolerated higher concentrations of compound **1** than the wild type strain (Figure 2B).

MmpL3 is a transmembrane protein that belongs to the resistance, nodulation, and division (RND) superfamily [32,33]. It is an essential protein that depends on the PMF for the transport of mycolic acids in the form of trehalose monomycolates (TMMs) across the cell membrane. MmpL3 acts as a flippase for TMMs at the inner membrane [15]. Many MmpL3 inhibitors with diverse chemical scaffolds have been described [34,35], such as a 1,2-diamine, SQ109 [18,36], the pyrrole derivative BM212 [37], the adamantyl urea AU1235 [17], indolamides [38,39], a spirocycle [40], a piperdinol-containing compound, PIPD1 [41], and a guanidine-based compound [42]. These molecules have been shown to specifically bind to MmpL3 and thus block its activity [15,43]. The crystal structure of *M. smegmatis* MmpL3 has been determined and it has been shown that inhibitors with various chemical scaffolds bind to the same pocket in the proton translocation channel [44].

### 2.5. Molecular Docking of the MmpL3 Complex with Compound **1**

**Figure 4:**
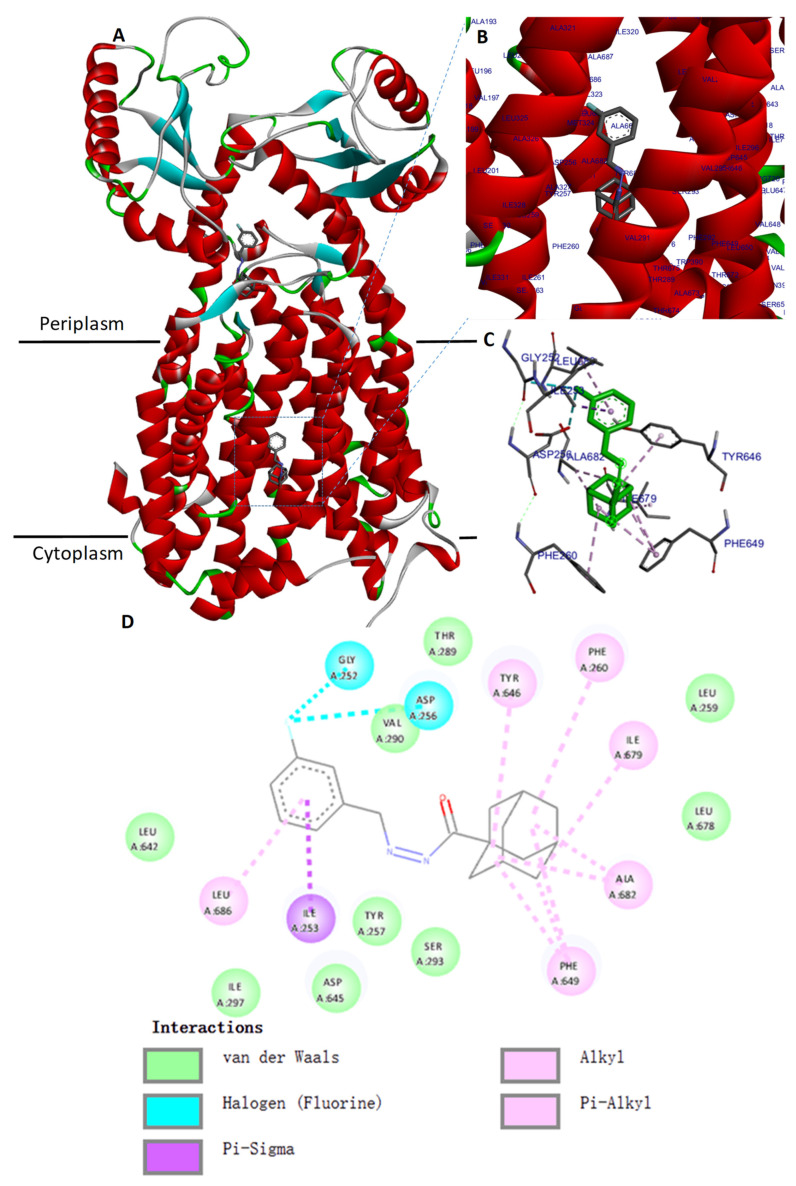
The docking site of compound 1 with *M. smegmatis* MmpL3. (**A**) Representation of an X-ray structure of the *M. smegmatis* MmpL3 (PDB ID 6AJH) with docked ligand compound **1** in the binding pocket of the transmembrane domain and in the cavity of the periplasmic domain. Compound **1** is depicted in stick representation in grey. (**B**,**C**) Enlargement of the compound **1**-binding pocket. (**D**) 2D interaction diagram for the complex MmpL3-compound **1** drawn using Discovery Studio Visualizer and showing amino-acid residues involved in the interaction. The adamantane moiety of compound **1** interacts with residue Phe649.

We evaluated the mode of interaction of compound **1** using a molecular docking simulation with MmpL3 from *M. smegmatis* (PDB ID 6AJH) [44]. The binding affinity score of the best binding conformation of compound **1** was −10.01 kcal/mol. We validated the docking protocol by redocking the ligand AU1235 with the MmpL3 binding site, resulting in a binding affinity score of −9.11 kcal/mol. The binding mode of compound **1** with MmpL3 is similar to that of other antituberculars that target MmpL3, SQ109 and AU1235, in the same pocket inside the proton-translocating channel, in the transmembrane region, as demonstrated in the crystal structure of MmpL3 from *M. smegmatis* [44] (Figure 4A–C). We then examined the interaction between compound **1** and MmpL3 using Discovery studio Analyzer. Phe649 was identified to have three alkyl interactions with the adamantane moiety of compound **1** (Figure 4D). The two pairs of hydrophilic residues (Asp256-Tyr646 and Asp645-Tyr257), previously shown to have an important role in proton translocation in MmpL3 [44,45], were also found to interact with compound **1**.

Surprisingly, another predicted binding site for compound **1** was identified during the molecular docking against MmpL3 from *M. smegmatis* with a binding affinity score of −8.77 kcal/mol. This docking pose was inside the cavity of the periplasmic domain that would allow entry or exit of specific molecules such as lipids [44,46]. This cavity is known to have a role in the binding and the transfer of TMMs, but not as a drug binding pocket.

The docking was also performed on the recently published Cryo-EM structure of *M. tuberculosis* MmpL3 (PDB ID: 7NVH) [47]. Contrary to what we assumed, compound **1** did not bind the drug binding pocket inside the proton-translocating channel, but inside the periplasmic cavity, and the best binding affinity score was −9.14 kcal/mol for this pose. The docking results of compound **1** against *M. tuberculosis* MmpL3 are given in the supporting information (Figure S4). Likewise, redocking the compound AU1235 with the Cryo-EM structure of MmpL3 from *M. tuberculosis* has not placed this molecule into the drug binding pocket but inside the periplasmic cavity in the same way as for compound **1**.

Until recently, docking of the MmpL3 inhibitors was performed on the *M. tuberculosis* MmpL3 homology model. The diverse inhibitors of MmpL3 previously identified are known to target the proton translocation path in the channel of transmembrane segment of MmpL3 [35,44,48]. However, some of these inhibitors could have multiple targets, such as BM212 that binds both MmpL3 and the transcriptional regulator of ethionamide EthR2 [49]. Other co-structures of the *M. tuberculosis* MmpL3 with inhibitors should be realized in order to confirm if an inhibitor could also bind to several places of the protein MmpL3. Various conformational states of MmpL3 have been identified during the lipid transport [50]. This diversity of conformational state could affect the docking result for obtaining an inhibitor that fit well into the binding site.

### 2.6. Measurement of the Mycobacterial Proton Motive Force

MmpL3 activity is linked to the proton motive force (PMF) of the inner membrane and a number of MmpL3 inhibitors have been shown to disrupt the membrane potential [51], whereas others do not [29]. We thus investigated whether compound **1** could affect the membrane potential (Δψ) using the fluorescent potentiometric dye DiSC3(5) (3,3′-dipropylthiadicarbocyanine iodide). Valinomycin and CCCP were used as controls.

**Figure 5:**
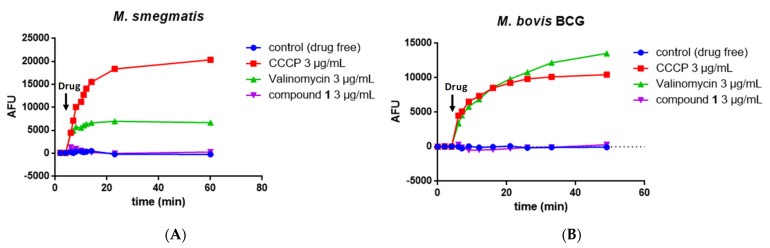
Effect of compound 1 on the mycobacterial transmembrane potential. Fluorescence of DiSC3(5) over time following the addition of DMSO (negative control), CCCP, valinomycin, or compound **1** to *M. smegmatis* (**A**) and *M. bovis* BCG (**B**). Membrane depolarization was measured using the membrane potential-sensitive fluorescent dye DiSC3(5), measured at λex 622 nm/λex 670 nm.

Valinomycin is a K^+^ ionophore that specifically dissipates the electrical potential (Δψ) component of the PMF in the presence of exogenous K^+^. The fluorescence intensity of DiSC3(5) increased in the presence of valinomycin and K^+^ in *M. smegmatis* (Figure 5A) and *M. bovis* BCG (Figure 5B). CCCP is a H^+^ ionophore that dissipates the proton gradient and thus destroys the PMF and uncouples oxidative phosphorylation. The fluorescence intensity of DiSC3(5) also increased markedly in the presence of CCCP in both *M. smegmatis* and *M. bovis* BCG (Figure 5A,B).

The addition of **1** did not significantly change the fluorescence intensity of DiSC3(5)-treated *M. smegmatis* (Figure 5A) or DiSC3(5)-treated *M. bovis* BCG (Figure 5B). 

The PMF is composed of the membrane potential (Δψ) and transmembrane proton gradient (ΔpH). We thus investigated the potential effect of **1** on the ΔpH across the inner membrane using a pH-sensitive fluorescent dye BCECF-AN. In contrast to the positive control CCCP or nigericin, the addition of compound **1** at 1 and 10 µg/mL did not disrupt the proton gradient across the inner membrane (Table 5). Overall, these results support that compound **1** does not disrupt the PMF.

### 2.7. Compound **1** Shows No Cytotoxicity

**Figure 6:**
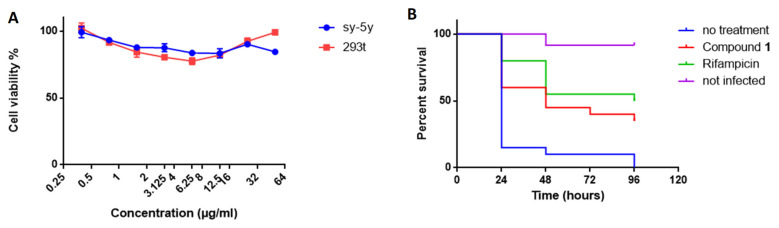
Assessment of cytotoxicity and *Galleria* treatment with compound **1.** (**A**) Cytotoxicity of compound **1** evaluated over a wide range of concentrations (50–0.2 μg/ml) against two mammalian cell lines (Sh-SY5Y and HEK 293T) by MTT assay. (**B**) Kaplan–Meier survival curves of *Galleria mellonella* larvae infected with *M. bovis* BCG and treated 2 h later with vehicle (6% DMSO) as a negative control, rifampicin as a positive control, and compound **1**.

We evaluated the cytotoxicity of compound **1** against two types of human cells lines, SH-SY5Y and HEK 293T, using the tetrazolium reduction assay (MTT). Compound **1** did not show any significant cytotoxicity up to a concentration of 50 μg/mL (Figure 6A).

The ADME prediction of compound **1** was performed using the SWISS ADME database [52]. The lipophilicity of compound **1** was calculated to have a consensus LogP of 3.70. Water solubility was calculated to have a LogS (ESOL model) of −4.07. Pharmacokinetic data predicted high gastrointestinal absorption and compound **1** obeys Lipinski’s Rules, with no violations.

### 2.8. Compound **1** Protects Galleria Mellonella Larvae against M. bovis BCG Infection

We did not have the possibility to use the mouse model to test the antibacterial efficacy of compound **1** in vivo. Thus, we used the larvae of the insect *Galleria mellonella* as an alternative model [53,54]. Larvae were infected with a lethal dose of *M. bovis* BCG and then treated with rifampicin or compound **1** and survival over 96 h was compared to that of the untreated control. The dose for the treatments was 10 mg/kg of body weight for both rifampicin and compound **1**, which is within the same order of magnitude as the dose used to treat mice with rifampicin [55]. The compounds were dissolved in 6% DMSO. A group of larvae was treated with a solution of 6% DMSO as a control.

Untreated larvae infected with *M. bovis* BCG (treated with vehicle) showed a survival rate of 10% after 48 h, whereas rifampicin treatment (positive control) resulted in 55% survival after 48 h (Figure 6B). Compound **1** also significantly improved survival, as the observed survival rate was 45% after 48 h. At the latest time point of 96 h, the untreated group showed a survival rate of 0%, whereas rifampicin treatment rescued larvae from *M. bovis* infection, with 50% survival, as did treatment with compound **1**, with 35% survival. The median survival time was less than 24 h in the untreated group, 48 h for the compound **1** group, and 96 h for the rifampicin group. The differences between the survival curves were statistically significant by the log-rank test (*p* < 0.0001). Thus, compound **1** showed protective effects in vivo against an *M. bovis* infection over time in *Galleria mellonella*.

## 3. Materials and Methods

### 3.1. Drugs and Reagent Preparation 

The commercial drugs isoniazid, rifampicin, and linezolid were purchased from Sigma, bedaquiline and nigericin from TargetMol Chemicals Inc, Boston, USA, and valinomycin from Shanghai Acmec Biochemical Co., Ltd, Shanghai, China. Antibiotic solutions were prepared at a concentration of 5 mg/mL in dimethyl sulfoxide (DMSO) from Sangon Biotech Co., Ltd, Shanghai, China. 

The 52,000 compounds from Topscience Co., Shanghai, China, to be screened were dissolved in 100% DMSO. Lead compound **1** showed 98.6% purity in HPLC analysis. Resazurin sodium salt powder (BBI Life Sciences, Shanghai, China) was prepared at 0.01% (*w*/*v*) in distilled water, filter sterilized, and stored at 4 °C for up to two weeks.

### 3.2. Strains and Growth Condition

*M. bovis* BCG (strain 1173P2), *M. marinum* (ATCC BAA-535), *M. smegmatis* mc2155, *M. abscessus* (ATCC 19977), *M. aurum*, *M. avium* (ATCC 25291), and *M. tuberculosis* H37Rv (ATCC 27294) were obtained from the collection of the Institut Pasteur. *M. tuberculosis* clinical isolates, including three susceptible, three Rif-resistant and one MDR strain were collected from hospitals in China. Mycobacteria were cultured in Middlebrook 7H9 broth containing 10% (*v*/*v*) ADC enrichment (albumin, dextrose, catalase; Becton Dickinson, Franklin Lakes, USA), 0.05% glycerol (Sangon Biotech, Shanghai, China), and 0.05% Tween 80 (Sangon Biotech, Shanghai, China), or Middlebrook 7H11 agar medium supplemented with 10% (*v*/*v*) OADC enrichment (oleic acid, albumin, dextrose, catalase; Becton Dickinson, Franklin Lakes, USA). All mycobacteria were cultured under aerobic conditions at 37 °C, except for *M. marinum*, which was cultured at 30 °C. *E. coli* ATCC 25922 and *S. aureus* ATCC 25923 were cultivated at 37 °C in LB medium.

### 3.3. Screening and MIC and MBC Determination

Screening of a chemical library composed of 52,000 compounds from Top Science was performed at the People’s Hospital of Shenzhen. The small chemical compounds were screened at 2 μg/mL (which corresponds to an average concentration of 5 to 6 μM) in the presence of 0.15 µg/mL linezolid. Each compound was prepared in 50 µL Middlebrook 7H9 broth in a 96-well plate and a 50 µL aliquot of *M. bovis* BCG suspension then added to each well. The plates were incubated at 37 °C for two weeks to allow growth of the bacteria. The antimycobacterial activity of compounds was determined using the resazurin-reduction assay. A change in color from blue to pink indicated bacterial growth. DMSO was used as a positive control to define growth of the bacteria without any compound (100% viability) and rifampicin was used as a negative control to kill all bacteria (0% viability). Primary hits were filtered using an activity cut-off (MIC < 4 µg/mL against *M. bovis* BCG) and by selecting for drug-like properties.

Determinations of MICs were performed by the microdilution method in 96-well plates, with each well containing 100 µL bacterial suspension with a serial dilution of the compound. The MICs were defined as the lowest concentration of compound that inhibited bacterial growth and thus prevented the change in color of resazurin. Pink wells indicated bacterial growth and blue wells no bacterial growth. Isoniazid and rifampicin were used as standards.

MBCs were determined by transferring 100 µL from each of the wells from the MIC assays (starting from the MIC for each compound) onto 7H11 plates. Plates were incubated at 37 °C for two days (*M. smegmatis*) or three weeks (*M. bovis*). The MBC was defined as the first plate (lowest concentration) yielding no growth.

### 3.4. Dose–Response Curves

*M. smegmatis* and *M. bovis* BCG were grown in 7H9 medium to an optical density at 600 nm of 0.5 to 1.0 and the cultures then diluted to an OD600 of 0.05 in 7H9 medium. Compounds were tested at concentrations between 0.01 and 5 µg/mL. The controls included DMSO and 2 µg/mL rifampicin. The antimicrobial potency of the compounds was evaluated at 600 nm by comparison with DSMO-treated (drug free, 100% growth) and rifampicin-treated bacterial suspensions (0%). Dose–response assays were conducted in triplicate and the curves created using GraphPad Prism 6 software (San Diego, CA, USA, version 6.0.1).

### 3.5. Kill Kinetics 

*M. smegmatis* and *M. bovis* BCG were cultured in 7H9 broth to log phase (10^5^–10^6^ CFU/mL), various concentrations of compound **1** in 7H9 (final concentration 0.5% DMSO) added, and the cultures incubated at 37 °C. The untreated control was 0.5% DMSO. The number of viable bacteria was determined by serial dilution and plating on Middlebrook 7H11 plus 10% vol/vol OADC. CFUs were counted after three days for *M. smegmatis* and 3 to 4 weeks for *M. bovis* BCG.

### 3.6. Generation and Analysis of Resistant Mutants

Resistant mutants were isolated by plating 10^9^ cells of *M. smegmatis* or *M. bovis* BCG (in 300 µL) onto plates containing 5 mL 7H11 medium with the 4×, 8×, and 16× liquid MIC of the compound. Resistant colonies were inoculated in 7H9 liquid medium containing the 2× MIC of the compound. The MICs of selected colonies were then determined by serial dilution. The MIC determinations were performed in triplicate for each strain of bacteria. The rate of resistance mutations was calculated as the ratio of CFU in the absence of the compound and CFU in presence of the compound.

### 3.7. Genome Sequencing and the Identification of Polymorphisms

The genomic DNA of selected mutants was extracted and purified using EZ-10 Spin columns from bacterial genomic DNA Mini-Prep Kits (Bio Basic, Amherst, MA, USA), sequenced, and analyzed for SNPs. Genome sequencing was performed by Sangon Biotech Co., Ltd, Shanghai, China. The genomes were sequenced using an Illumina HiSeq 2500 platform (Illumina, San Diego, CA, USA) and then quality-filtered and assembled using SPAdes Genome Assembler (St. Petersburg, Russia, version 3.12.0). The resulting reads were mapped to the *M. smegmatis* MC2 155 reference genome and mutations were identified using the Snippy pipeline (https://github.com/tseemann/snippy) (accessed on 16 July 2021).

The sequences assembled in the present study can be accessed through GenBank (NCBI) using the accession codes PRJNA766507.

SNPs were confirmed by PCR amplification and sequencing using primers MSMEG_0250-F and MSMEG_0250-R (Supporting Information Table S2).

### 3.8. Checkerboard Synergy Assay

Drug interactions between the identified compound and other antimycobacterial drugs were assessed with *M. bovis* BCG and *M. smegmatis* using the checkerboard microdilution method and resazurin assay as a viability marker.

Briefly, 50 µL 7H9 medium was distributed into each well of 96-well microdilution plates. Serial two-fold dilutions of each drug to at least double the MIC were prepared prior to testing. Compound 1 was serially diluted along the ordinate and the second drug along the abscissa. Each well was then inoculated with 50 µL of a bacterial inoculum of 10^5^ CFU/mL and the plates were incubated at 37 °C for seven days under aerobic conditions. The experiment was carried out in triplicate.

The fractional inhibitory concentration index (FICI) was determined in a 96-well plate and calculated by adding the FIC_A_ (MIC of compound A in the presence of compound B)/(MIC of compound A alone) and the FIC_B_ (MIC of compound B in the presence of compound A)/(MIC of compound B alone). Synergy was defined as an FICI value ≤ 0.5, while the values 0.5 < FICI ≤ 1 correspond to additivity; 1 < FICI ≤ 4 is indifferent; FICI > 4 indicate antagonism [56,57].

### 3.9. Docking Studies

Docking of compound 1 was carried out using the crystal structure of MmpL3 from *M. smegmatis* (PDB ID: 6AJH) obtained from the Protein Data Bank. This protein was considered as the target, and was prepared by adding hydrogens, calculated Gasteiger charges, and the complexed ligands were manually removed using the AutodockVina program. Compound **1**, considered as the ligand, was sketched using Marvin Sketch tools from ChemAxon (Budapest, Hongary) and converted into 3D using Discovery Studio Analyzer. The docking simulation was performed using Autodock Vina. A grid box was created with 50 × 50 × 50 points, with a resolution of 0.375 Å. The coordinates of the grid center were x 37.00, y 4.00, and z −22.00. The Lamarckian genetic algorithm was used to run 100 dockings. The docked conformations were ranked according to the docking free energy. Information was collected and ligand binding site atoms were visualized using Biovia Discovery Studio Analyzer. The same procedure was performed using AU1235 as the control ligand to validate the docking protocol. Then, compound **1** and AU1235 were docked in the same way against MmpL3 from *M. tuberculosis* (PDB ID: 7NVH) [47].

### 3.10. Measurement of the Mycobacterial Transmembrane Potential ΔΨ

Exponentially growing *M. bovis* BCG or *M. smegmatis* were harvested by centrifugation, washed once with buffer containing 5 mM Hepes, 5 mM glucose, and 0.05% Tween (pH 7.2), resuspended in the same buffer containing 2 μM DiSC3(5), and incubated for 2 h at 37 °C. Then, suspensions were dispensed into costar white flat-bottom 96-well plates (100 μl/well) and treated with compounds, CCCP, or valinomycin. Fluorescence was monitored (excitation wavelength, 622 nm; emission wavelength, 670 nm) on a Tecan Spark 10 M microplate reader at room temperature. Wells containing cells in the presence of DiSC3(5) and DMSO served as controls. All assays were performed at least twice (a representative assay is shown).

### 3.11. Measurement of the Mycobacterial Chemical Proton Potential ΔpH

*M. smegmatis* or *M. bovis* BCG were labeled with 20 µM of the fluorophore BCECF-AM (Invitrogen) in 0.1 M Hepes buffer, pH 7.0, for 30 min at 37 °C. Cells were pelleted by centrifugation and re-suspended in 0.1 M Hepes at various pHs (6.0–8.0) in the presence of 20 μM nigericin to generate a standard curve. Fluorescence emission (λem 525 nm) intensities of intracellular BCECF were measured following excitation at λex 488 and 440 nm in a Tecan Spark 10 M microplate reader. The effect on intracellular pH was studied after the treatment of *Mycobacterium* cells with various concentrations of compound 1 in 0.1 M Hepes at pH 6.8. DMSO was used as a negative control and CCCP and nigericin as positive controls.

### 3.12. Adme Prediction

An ADME (adsorption, distribution, metabolism, and excretion) prediction was performed in silico using the web tool SWISS-ADME (https://www.swissadme.ch) (accessed on 17 October 2022). 

### 3.13. Cytotoxicity

The cytotoxicity of compound 1 was assessed on two different mammalian cell lines (Sh-SY5Y and HEK 293T) by the MTT colorimetric assay using tetrazolium reduction. Cell suspensions were cultivated in RPMI-1640 medium supplemented with 10% fetal bovine serum (FBS) and incubated at 37 °C in a 5% CO_2_ atmosphere. The cytotoxicity assays were performed in 96-well microplates, adding 100 µL of culture with 4 × 10^4^ cells/mL to each well. On the next day, cells were treated with the compounds prepared in DMSO and serially diluted from 50 to 0.1 µg/mL. Each drug was tested in triplicate. The plates were examined after 48 h of incubation, according to the manufacturer’s instructions. The percentage viability was calculated by comparison with the control without treatment.

### 3.14. Infection of Galleria Mellonella and Drug Treatment

Larvae were purchased from Beijing KuoyeTianyuan Biotechnology C., Ltd, Beijing, China. Healthy, high-mobility, ~300-mg cream-colored larvae were used for the infection experiments. Injections into the hemocoel via the last left proleg were performed with a 10-μL Hamilton syringe. For infection, 10 µL of a *M. bovis* BCG (10^7^ CFU) suspension in PBS, 0.05% Tween was injected [53]. For treatment, 10 µL of compound (rifampicin or compound 1) diluted in 6% DMSO at a dose of 10 mg/kg was injected 2 h after infection (*n* = 20). The control group of infected larvae was injected with 10 µL 6% DMSO (*n* = 20) and the control group of uninfected larvae (*n* = 20) with 10 µL PBS, 0.05% Tween. Larvae were then incubated at 37 °C in Petri dishes (5 per box) in the dark without food.

Survival of the infected larvae (*n* = 20 per group) following treatment was monitored every 24 h for 96 h. Larvae were considered dead when they were unresponsive to touch. Kaplan–Meier survival curves were used to compare the distribution of survival of each group of larvae.

## 4. Conclusions

The emergence of drug-resistant TB is a major global health challenge. Controlling the disease will require new molecules that can be used in combination with approved drugs to improve and shorten treatment time. Here, phenotypic screening of a small-molecule chemical library allowed the identification of several molecules with anti-mycobacterial activity. Among them, we identified compound **1** as a probable inhibitor of MmpL3, with a new scaffold containing an adamantane moiety and a hydrazide–hydrazone moiety. Compound **1** showed antimycobacterial activity against *M. smegmatis*, *M. bovis* BCG, and *M. tuberculosis* H37Rv. It was also active against a panel of clinical drug-sensitive, Rif-R, and MDR *M. tuberculosis* strains, with MIC values of 0.2 to 0.4 µg/mL and moderately against the nontuberculous mycobacterial species *M. abscessus* (MIC value of 12.5 µg/mL).

Whole-genome sequencing of spontaneous mutants resistant to compound **1** allowed us to identify MmpL3 as the putative target of compound **1**. MmpL3 is an inner membrane protein that transports mycolic acids in the form of trehalose monomycolates (TMMs) across the membrane. We generated independent compound **1**-resistant mutants with a F649L mutation localized in the transmembrane segment of MmpL3, inside the proton-translocation channel. Amino acid changes at F649 in *M. smegmatis* correspond to F644 in *M. tuberculosis*, which have been reported previously for other MmpL3 inhibitors [13,31,44,58]. Molecular docking suggested that compound **1** could also binds within the binding pocket of *M. smegmatis* MmpL3 and interacts with the two Asp-Tyr pairs (Asp256-Tyr646 and Asp645-Tyr257) involved in proton translocation which allow the energy necessary for the substrate translocation [44,45], although the docking of compound **1** against the Cryo-EM structure of *M. tuberculosis* MmpL3 did not reveal an interaction within the binding pocket in the same way as in *M. smegmatis*, but only in the cavity at the center of the periplasmic domain. 

Moreover, the mode of action of MmpL3 inhibitors is debated because the activity of MmpL3 is also linked to the proton motive force (PMF) of the inner membrane. Several studies have proposed that certain inhibitors indirectly target MmpL3 and disrupt the membrane potential [51,58], whereas others do not [29]. If compound **1** targeted MmpL3, we also found that this compound did not affect the inner membrane PMF.

We show that compound **1** acts synergistically in combination with linezolid against *M. bovis* BCG and *M. smegmatis* but not against *M. tuberculosis*. An enhancer that increases the activity of linezolid could allow a reduction in the dose of linezolid during treatment and thus reduce adverse effects. Future works with other compounds showing similarity with compound **1** may provide useful information.

We investigated the effect of compound **1** on a *G. mellonella* larvae model of infection to evaluate its activity in vivo. Compound **1** showed no toxicity towards the larvae and a protective effect against *M. bovis* BCG. This is the first time that an inhibitor targeting MmpL3 has been used in *G. mellonella* larvae, validating the suitability of *G. mellonella* larvae as an inexpensive and efficient in vivo model for the characterization of new antimycobacterial compounds. Based on our results, we consider compound **1** to have promising therapeutic potential.

## Figures and Tables

**Table 1:** In vitro antibacterial activity of compound **1**. MICs and MBCs values from three independent determinations in µg/mL. 7H9 and 7H11 media were used for *Mycobacterium* species. LB medium was used for *S. aureus* and *E. coli*.

Bacterial Strains	MIC	MBC
*M. tuberculosis* H37Rv	0.2	n.d.
*M. bovis* BCG	0.3	0.6
*M. smegmatis*	1.5	3
*M. marinum*	12.5	n.d.
*M. abscessus*	12.5	n.d.
*M. avium*	>25	n.d.
*S. aureus*	>25	n.d.
*E. coli*	>25	n.d.

MIC: minimum inhibitory concentration, MBC: minimum bactericidal concentration, n.d.: no data.

**Table 2:** MICs of compound **1** against seven *M. tuberculosis* clinical strains. MICs in µg/mL. Different MIC values for the same strain come from independent experiments.

*M. tuberculosis* Clinical Strain	Strain Type	MIC
**S17**	Sensitive	0.2–0.39
**S23**	Sensitive	0.2–0.39
**S31**	Sensitive	0.2–0.39
**M9**	Rif-R	0.78
**M20**	Rif-R	1.56
**M34**	Rif-R	0.39–0.78
**M7**	MDR	0.2–0.39

**Table 3:** FICI (fractional inhibitory concentration index) values obtained for linezolid with compound **1** against *M. bovis* BCG, *M. smegmatis*, and *M. tuberculosis* H37Rv. The values were obtained in three independent experiments.

	* M. bovis * BCG	* M. smegmatis *	* M. tuberculosis *
Linezolid	0.50	0.28	0.68

**Table 4:** Polymorphisms identified in *M. smegmatis* resistant mutants.

	REF	ALT	Amino Acid Change	Impact	Gene Name	Description
**Mutant** **Δ1**						
**snp**	TTC	**C**TC	Phe649Leu	missense	MSMEG_0250	MmpL3
**snp**	CAC	CA**T**	His80His	synonymous		IS1 family transposase IS1S
**Mutant** **Δ2**						
**snp**	TTC	**C**TC	Phe649Leu	missense	MSMEG_0250	MmpL3
**snp**	AGA	AG**G**	Arg54Arg	synonymous		IS1 family transposase IS1S
**Mutant** **Δ** **3**						
**snp**	TTC	**C**TC	Phe649Leu	missense	MSMEG_0250	MmpL3

**Table 5:** pH outside refers to pH of external buffer. pH inside is the intracellular pH obtained using the BCECF-AN dye. Measurements of the fluorescence ratio (λex 488 nm/λex 440 nm) of BCECF were averaged (triplicates) and calibrated against a standard curve.

Drug	pH Outside	pH Inside	ΔpH
**DMSO**	6.80	7.20 (±0.01)	0.40 (±0.01)
**Nigericin 5 µg/mL**	6.80	6.76 (±0.01)	−0.04 (±0.01)
**CCCP 1 µg/mL**	6.80	6.91 (±0.01)	0.11 (±0.01)
**Compound 1 10 µg/mL**	6.80	7.13 (±0.02)	0.33 (±0.02)
**Compound 1 1 µg/mL**	6.80	7.19 (±0.01)	0.39 (±0.01)

## Data Availability

Not applicable.

## Supplementary Materials

The following supporting information can be downloaded at https://www.mdpi.com/article/10.3390/molecules27207130/s1, Figure S1: Intracellular calibration of the pH-sensitive dye BCECF-AN. Figure S2: Proof of molecular structure for compound **1** from proton nuclear magnetic resonance (1H NMR). Figure S3: HPLC trace of compound **1**. Figure S4: The docking site of compound **1** with *M. tuberculosis* MmpL3. Table S1: Mutation in *rpoB* in clinical strains used in the study. Table S2: Primers used in this study. Molecular formula strings file for compound **1** as data sheet in csv format. Table S3: Molecular formula strings.

## Abbreviations

CFU	colony-forming unit
DMSO	dimethyl sulfoxide
EMB	Ethambuthol
FBS	fetal bovine serum
FICI	fractional inhibitory concentration index
MBC	minimum bactericidal concentration
MDR	multidrug-resistant
MIC	minimum inhibitory concentration
MmpL3	mycobacterial membrane protein large 3
PZA	Pyrazinamide
Rif-R	rifampicin resistant
TMMs	trehalose monomycolates
