# Supplementary material for: A Hydrazine–Hydrazone Adamantine Compound Shows Antimycobacterial Activity and Is a Probable Inhibitor of MmpL3
Source: Molecules. 2022 Oct 21;27(20):7130. doi: 10.3390/molecules27207130 (PMC9610904; doi:10.3390/molecules27207130)
Supplement: Supplementary file 1 [file molecules-27-07130-s001.zip › molecules-1924330-supplementary.pdf]

Supporting Information

A hydrazine-hydrazone adamantine compound that target MmpL3 shows antimycobacterial activity

Julien Briffotaux[1,2*&], Yanji Xu[1,2&], Wei Huang[1,2&], Zhen Hui[2], Xiao Wang[1], Brigitte Gicquel[1,3£], Shengyuan Liu[1£*]

[1]Department of Tuberculosis Control and Prevention, Shenzhen Nanshan Center for Chronic Disease Control, 7 Huaming Road, Shenzhen, 518054, China.
[2]Bacteriology & Antibacterial Resistance Surveillance Laboratory, Shenzhen Institute of Respiratory Diseases, Shenzhen People's Hospital (The Second Clinical Medical College, Jinan University; The First Affiliated Hospital, Southern University of Science and Technology), No 1017 Dongmen North Road, Shenzhen, 518020, China.
[3]Mycobacterial Genetics Unit, Institut Pasteur, 25 Rue du Docteur Roux, 75724, Paris, France.
& Equal participation
£ Equal participation

*Corresponding Authors: Shengyuan Liu liushenglb@126.com
Julien Briffotaux julien.briffotaux@gmail.com

Table of contents

Figure S1. Intracellular calibration of the pH-sensitive dye BCECF-AN. S2
Figure S2. 1H-NMR spectrum of compound 1. S2
Figure S3. HPLC trace of compound 1. S3
Figure S4. The docking site of compound 1 with *M. tuberculosis* MmpL3 S3
Table S1. Mutation in rpoB in clinical strains Rif-R used in the study. S4
Table S2. Primers used in this study. S4
Table S3. Molecular formula strings. S4

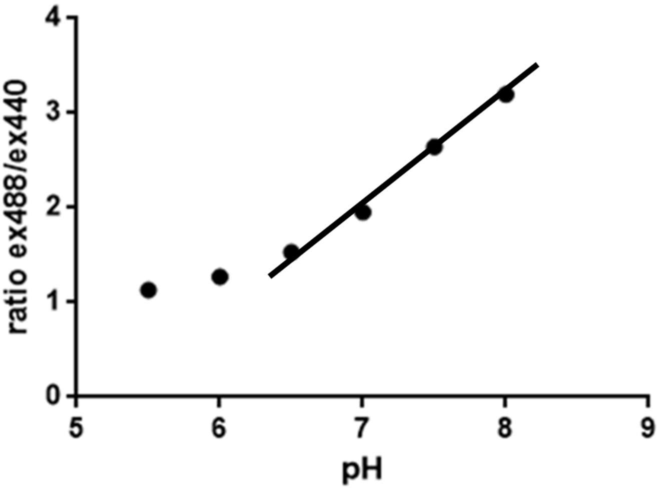

Figure S1. Intracellular calibration of the pH-sensitive dye BCECF-AN. Measurements of the fluorescence ratio (λex 488 nm/λex 440 nm) of BCECF in *M. bovis* BCG were averaged (triplicates). pHi calibration curve was sigmoidal with linear range in the physiological pH range of 6,5 - 8.

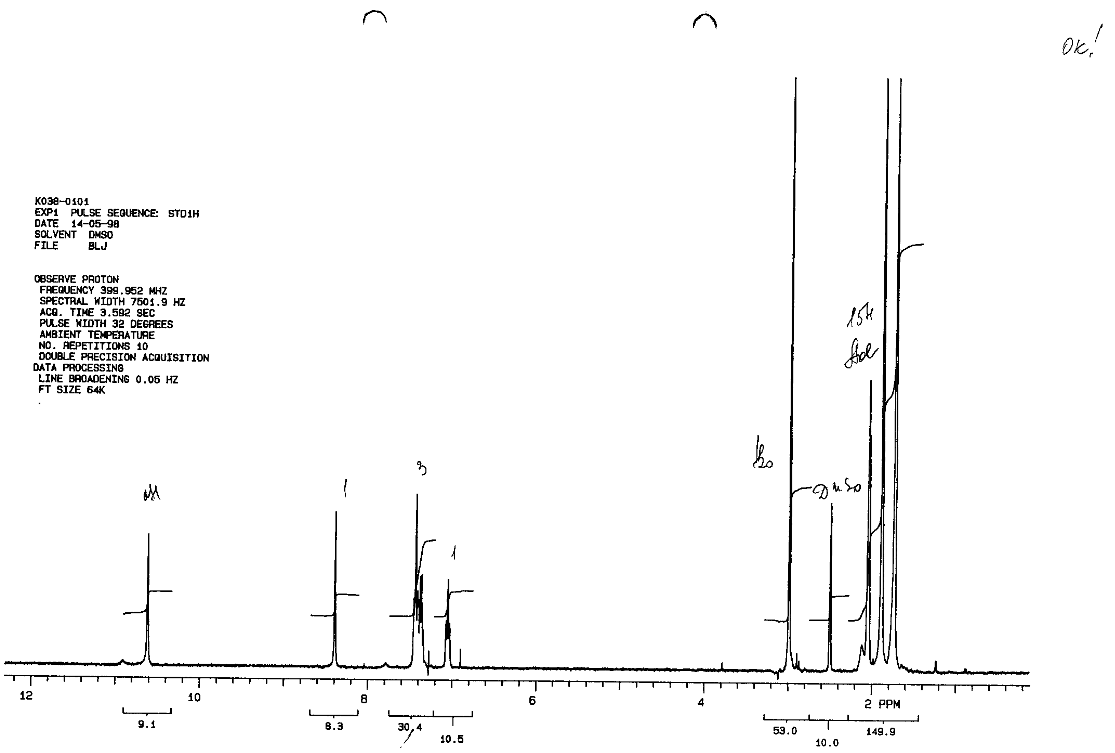

Figure S2. 1H-NMR spectrum of compound 1 (400 MHz).

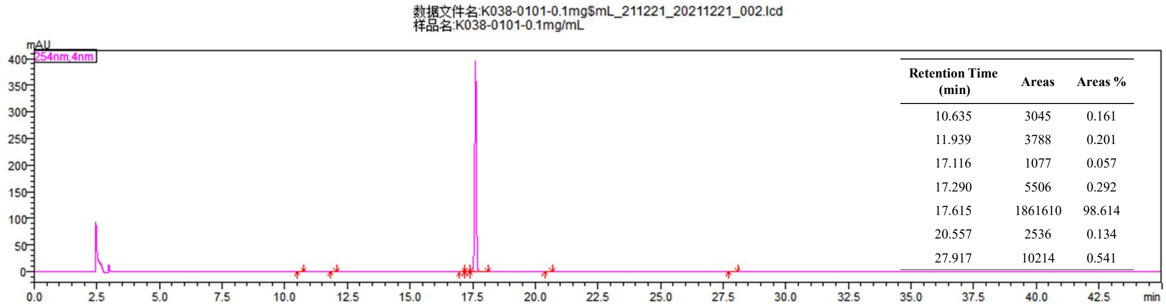

Retention Time (min)	Areas	Areas %
10.635	3045	0.161
11.939	3788	0.201
17.116	1077	0.057
17.290	5506	0.292
17.615	1861610	98.614
20.557	2536	0.134
27.917	10214	0.541

Figure S3. HPLC trace of compound 1. The purity of compound 1 is 98.6%. HPLC conditions: Column: Phenomenex Kinetex XB-C18, 5 µm, 100 Å, 4.6*250 mm. Mobile A: acetonitrile, mobile B: 2 mM ammonium formate; gradient: 0-10 min with 10-60 % A, 10-30 min with 60-95 % A, 30-40 min with 95 % A, 40-40.1 min with 95-10 % A, 40.1-45 min with 10 % A. Flow rate: 1 mL/min; column temperature: 35℃; detector: 254 nm; injection volume: 10 µL. Sample: 0.1 mg/mL in DMSO.

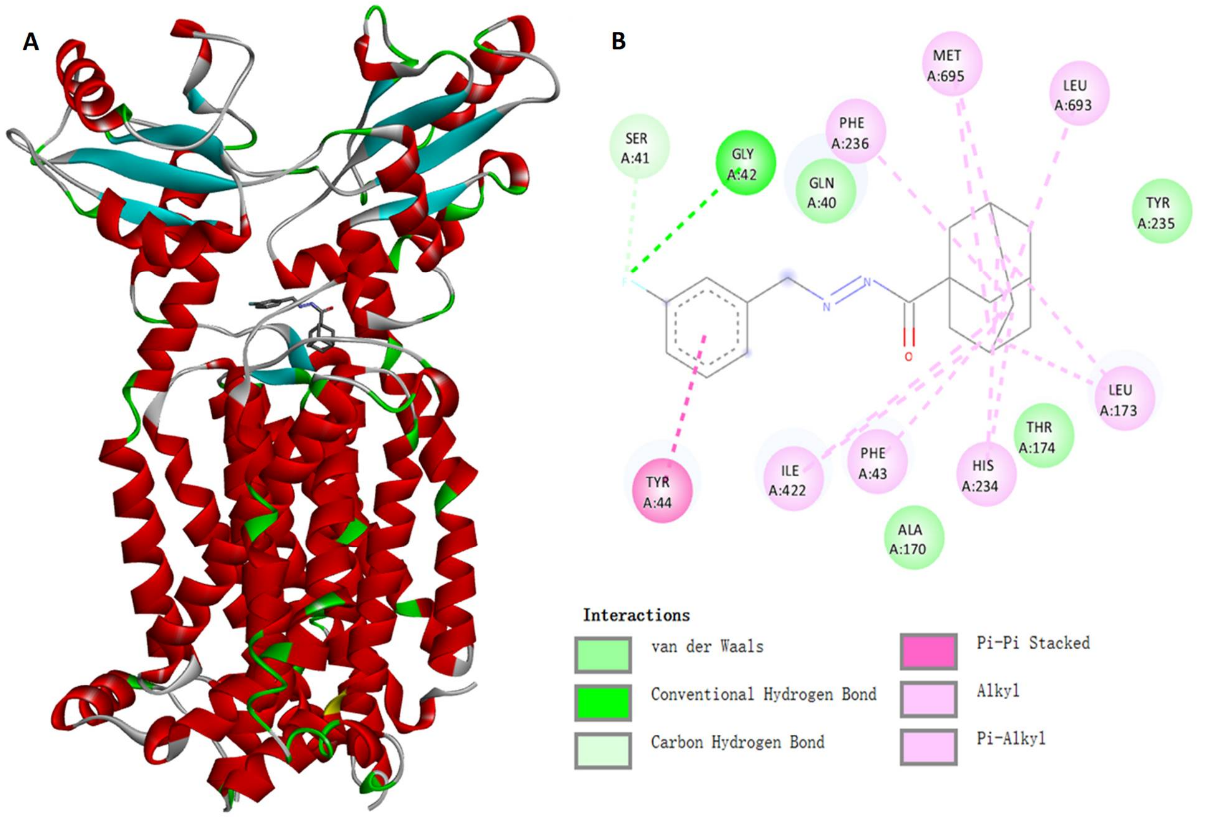

Figure S4. **The docking site of compound 1 with *M. tuberculosis* MmpL3.** A. Representation of an X-ray structure of the *M. tuberculosis* MmpL3 (PDB ID: 7NVH) with docked ligand compound **1** in the cavity of the protein. Compound 1 is depicted in stick representation in grey. B. 2D interaction diagram for the complex MmpL3-compound **1** drawn using Discovery Studio Visualizer.

**Clinical strain**	**Mutation in *rpoB***	
	Base	Amino Acid
**M9**	1590C>T;1714A>G	Ser531Leu; Ile572Val
**M20**	1590C>T	Ser531Leu
**M34**	1598T>C	Leu533Pro

Table S1. Mutation in *rpoB* in clinical strains Rif-R used in the study.

**Primers**	**5‘ to 3’ Sequence**
MSMEG_0250-F	ccttggagtttgtcgatc
MSMEG_0250-R	cggtggcattcctcaaca

Table S2. Primers used in this study.

Compound	SMILE	Molecular Formula	Molecular Weight
Compound **1**	Fc1cccc(c1)/C=N/NC(=O)C12CC3CC(C1)CC(C2)C3	$C_{18}H_{21}FN_2O$	300.4

Table S3. Molecular formula strings.
